# A spectroscopic dataset for known provenance and post-consumer textiles

**DOI:** 10.1038/s41597-026-07434-6

**Published:** 2026-05-22

**Authors:** Katarina E. Goodge, Alexander K. Landauer, Cecelia J. Vederman, Amanda L. Forster

**Affiliations:** 1 National Institute of Standards and Technology, Material Measurement Laboratory, 100 Bureau Drive, Gaithersburg, MD 20899 USA; 2 Georgetown University, Institute for Soft Matter Synthesis and Metrology, 3700 O St NW, Washington, DC 20057 USA; 3 Montgomery College, 20200 Observation Drive, Germantown, MD 20876 USA

## Abstract

Near infrared (NIR) spectroscopy is a rapid, non-invasive technique often used for chemical bond structure identification, making it a prime candidate for feedstock identification and validation for industrial processes involving polymers. It has rapidly gained popularity in the textile industry; however, the availability of high-quality, known provenance NIR data for textile fibers and fabrics is limited. Applying NIR to answer questions such as fiber classification or polymer blend identification typically requires the use of models or algorithms. The underpinning data for these models is typically in proprietary libraries or self-built databases; thus, benchmarking model performance across the industry is challenging. Here, a new dataset is presented to address this challenge. The dataset contains data on textile specimens for applications including fiber content classification, systems and software development, and validation of textile sorting systems. The data repository includes directories for benchtop NIR spectral data, handheld NIR spectral data, and fabric-scale microscopy images.

## Background & Summary

Chemical composition can be determined by a number of techniques such as Fourier Transform Infrared (FTIR) spectroscopy, Near-Infrared (NIR) spectroscopy, Raman spectroscopy, Nuclear Magnetic Resonance (NMR) spectroscopy, and Mass Spectrometry (MS). NIR spectroscopy is a noninvasive, nondestructive, rapid, in-line optical material characterization technique used in many industries, such as pharmaceuticals or plastics production. NIR sensors are often favored for high-throughput industrial applications because of their non-contact modality, which makes them easy to integrate into fully automated systems^1^. NIR has also previously been used in fiber identification^2–6^ for historical garments^7^, forensic applications^8,9^, quality control^10–14^, and emerging fields such as textile recycling^15–20^, demonstrating its versatility in characterizing textile materials. In a recent workshop geared towards reclaiming and reshoring textile production to the United States^21^, participants highlighted identification of fiber type using NIR for feedstock validation to be a challenge and a barrier to scale up of this nascent industry.

Studies using NIR data often focus on building and testing models to perform certain actions, such as classification and quantification of fiber identity. Many of these models are based on machine learning (ML) algorithms, which typically require extensive data inputs and are limited by the availability and quality of the input data. To date, libraries of NIR spectra for textiles have limited scope, may be inaccessible due to the use of proprietary information, or are otherwise not broadly available to the community. Datasets that are more broadly usable by the community follow open data guidelines such as the Findable, Accessible, Interoperable, and Reusable (FAIR) data principles^22^. Thus, to streamline modeling efforts, data needs to be collected and released in a high-quality, well-validated, open-source, and accessible manner to provide the underpinnings of these models and to compare model results.

A standard test method for fiber identification via NIR does not currently exist, further complicating efforts at comparison. The American Association of Textile Chemists and Colorists (AATCC) Test Method 20^23^ describes Fourier transform mid-IR spectroscopy, and ASTM Standard E1790-00^24^ describes NIR spectroscopy as a general methodology. With the diverse spectral ranges of assorted spectrometers combined with the varied quality and form factor of fabric samples, it is difficult to meaningfully compare different model results found in literature. Using one well-validated dataset as a basis for model comparison enables performance benchmarking and meaningful cross-comparison.

**Fig. 1:**
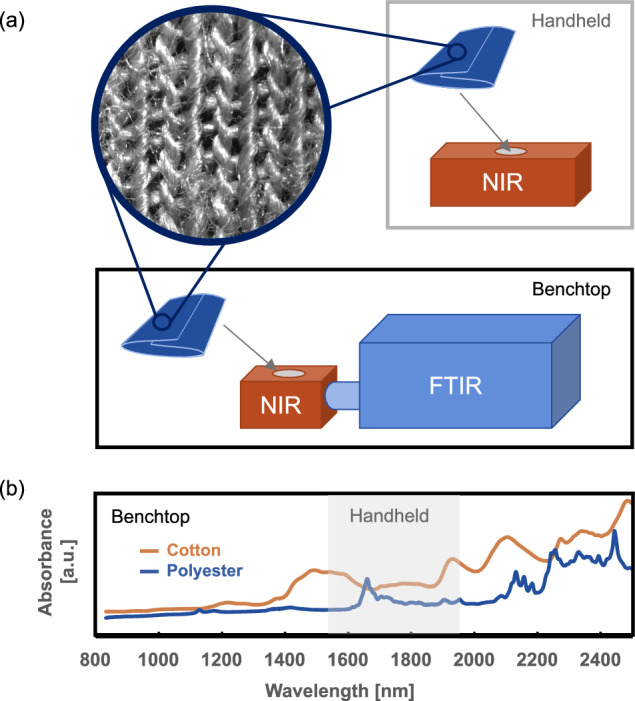
Data collection for fabric specimens. (**a**) Schematic of NIR spectroscopy measurement on handheld and benchtop instruments. The fabric specimen is folded to obtain six layers of thickness and positioned over the sampling window. Inset shows a microscopic image of an example knit fabric specimen. (**b**) Example NIR spectra of cotton and polyester fabrics with the handheld range (1550 nm to 1950 nm), shaded, and benchtop range (833 nm to 2500 nm).

As a first step towards addressing industry’s need for reliable, open-source data for model development and comparison, we introduce a dataset called Near-Infrared Spectra of Origin-defined and Real-world Textiles, or NIR-SORT. It aims to supply high-quality spectra of known provenance fabrics as well as real-world post-consumer fabrics. The data collection is diagrammed in Fig. 1. The data, with associated metadata and documentation, include benchtop NIR-FTIR spectral data (Fig. 1b), handheld NIR spectral data (Fig. 1b), and fabric-scale microscopy images (Fig. 1a). We describe the dataset framework for which version 1.0 and 2.0 has been released with a sample library of 113 fabrics and 61 fiber specimens. Future versions will provide additional instruments, spectroscopic techniques, and fabric samples. To improve accessibility, interoperablity and reusablity, the dataset consists of experimental metadata, machine data exported in common, widely used, or open formats, and example post-processed data. We believe this dataset is a foundation for benchmarking many analyses and models. We welcome suggestions and feedback from the community on this dataset. For future versions we will solicit external data contributions to improve the breadth and depth of FAIR NIR data available for textiles.

## Methods

### Materials

All specimens were taken from purchased fabrics. The origin-defined, or known provenance, fabrics were acquired from Testfabrics, Inc. (West Pittston, PA, USA) and were labeled Source A, known dyes, or Source B, undyed. Fabric specifications such as fiber content, fabric structure, dye type, finishes, manufacturer’s product identification code, and manufacturer’s lot identification code were provided by the manufacturer and can be found in SampleInformation.csv. The real-world, post-consumer garments were acquired from a local secondhand store and labeled Source C. The real-world, pre-consumer fabrics were obtained from sources such as production samples or swatch decks and labeled Source D. Fabric specifications such as fiber content and fabric structure were observed from the garments, e.g., their care label, and can be found in SampleInformation.csv. Manufacturer information is unknown unless otherwise specified. Fiber content represented in NIR-SORT 2.0 is summarized in Table 1.

**Table 1:** Summary of sample library fiber content as of February 2026.

Fiber(s)	Source A	Source B	Source C	Source D	Total
**Single Component**					
Cotton	33	4	5	0	42
Nylon	9	3	0	0	12
Polyester	4	5	2	0	11
Rayon	3	1	0	0	4
Wool	3	1	0	0	4
Acrylic	1	0	0	0	1
**Multicomponent**					
Cotton/Polyester	57	5	3	4	69
Cotton/Rayon	0	0	0	1	1
Cotton/Spandex	0	1	0	0	0
Nylon/Spandex	0	1	0	0	0
Nylon/Rayon/Spandex	0	0	0	1	1
Nylon/Rayon/Wool	0	0	0	1	1
Polyester/Spandex	0	1	0	0	0
Polyester/Cotton/Rayon	0	0	1	3	4
Polyester/Cotton/Spandex	0	0	0	1	1
Polyester/Rayon/Spandex	0	0	0	1	1
Total	110	22	11	12	174

See SampleInformation.csv in the dataset repository for more sample metadata.

### Sample Preparation

Specimens were measured in form specified in Column O of SampleInformation.csv. All specimens were conditioned, and all experiments were conducted under ambient laboratory conditions. Temperature was in the approximate range [23, 26] ^∘^ C, and relative humidity was in the approximate range [14, 46]% humidity. See daily average test conditions files in the corresponding folders (e.g., TestConditions_N.xlsx in the Benchtop_N\ directory). For NIR spectroscopy, each fabric specimen, approximate dimensions of 10 cm by 10 cm, was folded such that a total of six layers of fabric were stacked and positioned on the sampling window. Six layers of fabric minimizes light transmission without sacrificing sample handling. However, similar reflectance counts can be achieved with one layer of fabric by covering the specimen with a backing material. See Figure S1 in the Supplemental Information for performance comparisons of aluminum foil, buffed metal, black rubber, and two grayscale reflectance standards. For NIR-SORT 2.0 and future versions, specimens that were size-limited, i.e., insufficient fabric to stack six layers on the sensor, were measured with aluminum foil as backing material and denoted as such in the corresponding metadata files. Each replicate was measured on nonoverlapping weft and warp yarns such that each measurement is an independent sampling of the yarns, i.e., no two measurements contain the same set of yarns. For optical microscopy, each fabric specimen was laid flat as a single layer, centered under the objective lens, and held in place by stage clips. All instruments, for spectroscopy and microscopy, were warmed up for a minimum of 30 minutes before collecting measurements.

### NIR Spectroscopy

Specimens from the NIR-SORT sample library were measured via diffuse reflectance spectroscopy on a NIR-FTIR benchtop spectrometer (Thermo Fisher Scientific Inc., Madison, WI, USA) labeled Benchtop_N, a dispersive NIR handheld spectrometer (Matoha Instrumentation Ltd., London, UK) labeled Handheld_F, and a second dispersive NIR handheld spectrometer (Sortile Inc., New York City, NY, USA) labeled Handheld_S. Instrument information and measurement parameters for each instrument are provided in Table 2. Benchtop_N is a Nicolet is50 FTIR with an integrating sphere module attached to the main system on the left side, as shown in Fig. 1a. The operator must choose the integrating sphere in the instrument software and insert the Calcium Fluoride (*CaF*_2_) beamsplitter to access the NIR-FTIR module, which routes the light path through the main instrument to the integrating sphere. Beam alignment was performed at the beginning of every measurement session. Background measurements were collected before every sample at the same resolution and 128 scans. Each sample was measured as an interferogram and converted to an absorbance or reflectance spectrum by Fourier transformation within the instrument’s proprietary software. No corrections such as for environmental factors were applied. For Handheld_S, each sample was measured as reflectance counts and converted to absorbance, described in detail in the Technical Validation section. Data from Benchtop_N were measured in wavenumbers and converted to wavelength whereas Handheld_S and Handheld_F were measured in wavelength and can be converted to wavenumbers. Data was saved according to the prescribed file naming convention in comma separated value (.csv) files. For ease of use and differing preferences of potential dataset users, all combinations of *x* and *y* units are provided in the dataset either directly or via the Utility Scripts and are demonstrated in Fig. 5.

**Table 2:** Near Infrared Spectroscopy instrument information and measurement parameters for benchtop and handheld NIR instruments.

Parameters	Benchtop_N	Handheld_F	Handheld_S
**Instrument information**			
Instrument name	Thermo Fisher Nicolet iS50 FTIR, Integrating Sphere	Matoha FabriTell	Sortile
Detector	Indium Gallium Arsenide (InGaAs)	Indium Gallium Arsenide (InGaAs)	MEMS Fabry-Perot Interferometer
Crystal material	Sapphire	—	—
Reference material	Internal, Diffuse Gold	External, Fluorilon grayscale standards, 99% reflectance	
**Measurement parameters**			
Wavenumber range	3999.706 cm^−1^ to 11999.120 cm^−1^ (step size 1.928 cm^−1^)*	6450 cm^−1^ to 5130 cm^−1^**	6450 cm^−1^ to 5130 cm^−1^**
Wavelength range	833 nm to 2500 nm**	1550.0 nm to 1950.0 nm (step size ≈ 3.2 nm)*	1550.0 nm to 1950.0 nm (step size ≈ 3.2 nm)*
Resolution	4.000 cm^−1^	approx. 18 nm	15 nm to 21 nm
Y-values	Absorbance* & % Reflectance*	Absorbance** & Reflectance counts*	Absorbance** & Reflectance counts*
Number of scans	128	4	30
Number of replicates (nominal)	7	7	7

* as collected.

** converted.

### Optical Microscopy

**Fig. 2:**
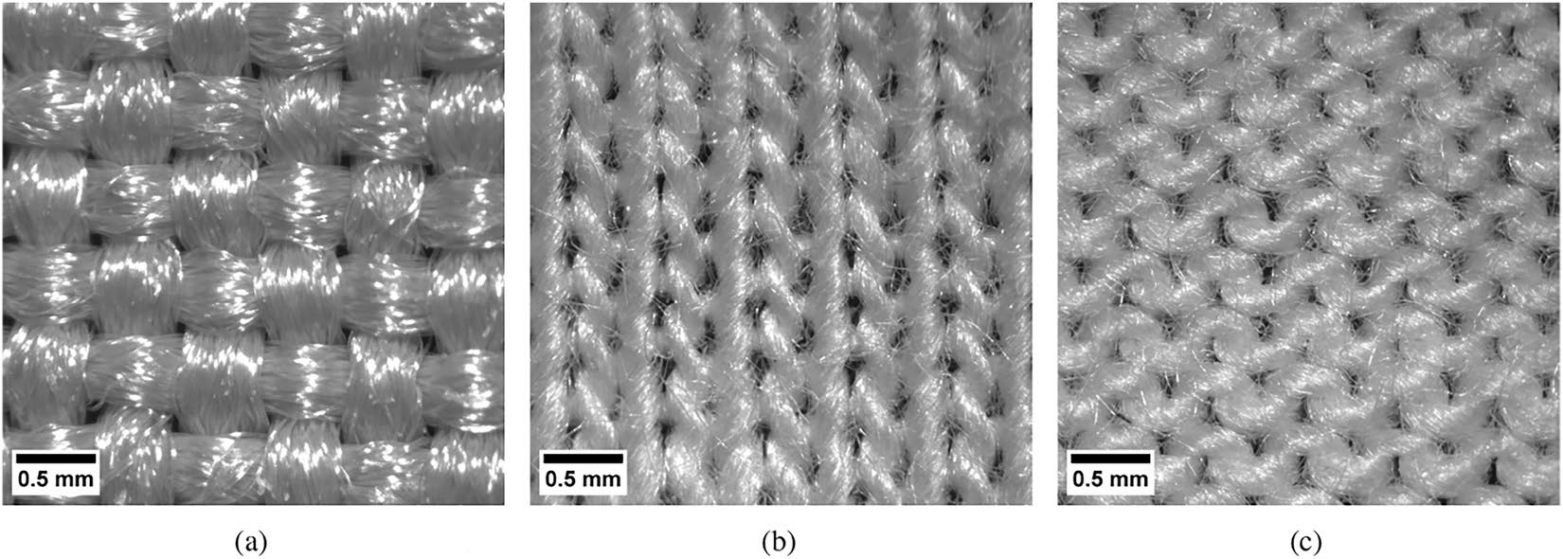
Examples of optical microscope images. Snapshots have been cropped for publication. (**a**) woven polyester, (**b**) knit polyester/cotton blend, front face, (**c**) knit polyester/cotton blend, back face.

For sample library cataloging and visual analysis, fabrics from NIR-SORT 1.0 were imaged with a monochromatic 8.9 MPx BlackFly S BFS-U3-89S6M camera (Teledyne FLIR LLC, Wilsonville, OR, USA) mounted to a stereo-ocular inspection microscope (unknown manufacturer). The entire fabric sample library for NIR-SORT 2.0 and future versions are imaged with a color 5.0 MPx BlackFly S BFS-U3-50S4C-CS camera (Teledyne FLIR LLC, Wilsonville, OR, USA) mounted on the same stereo-ocular microscope. Select fiber specimens were imaged with a polarized 5.0 MPx BlackFly S BFS-U3-51S5PC-C camera (Teledyne FLIR LLC, Wilsonville, OR, USA) mounted on the same stereo-ocular microscope. Camera and microscope settings (e.g., focus, exposure) were adjusted to obtain a clear image, a snapshot was taken using the Spinnaker SDK software (Windows), viewed using the SpinView 4.0.0.116 software (Windows) provided by the camera manufacturer, and saved as a .bmp or .tiff file. Example snapshots showing different fabric types are shown in Fig. 2.

## Data Records

**Fig. 3:**
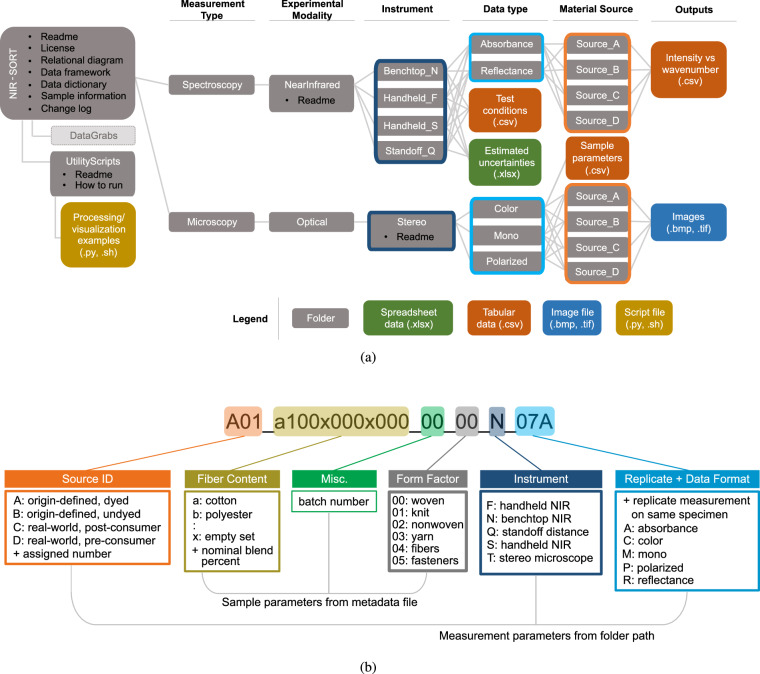
NIR_SORT dataset framework as described by the (**a**) folder structure and (**b**) file naming convention. Folders, denoted by grey fill and white font, are organized by measurement type, experimental modality, instrument, data type, and material source. File types are differentiated by fill color. “Spreadsheet data” is green, “tabular data” is orange, “image file” is blue, and “script file” is yellow. The DataGrabs\ folder appears with a lighter grey fill and dotted perimeter to denote it as a “phantom” folder which is created once the data_collection_from_folders.[.py |.sh] script is run. The file naming convention contains information from sample and measurement parameters. See DataFramework.txt for more details.

As described in the Methods section, data were collected from four primary experimental sources: benchtop NIR-FTIR spectrometer, two handheld NIR spectrometers, and stereo microscope. Primary reduced, or transformed, data from the instruments are stored in the .csv format in the data repository. The data framework is structured by a top-level directory and subfolders, schematically described in Fig. 3. The top-level directory contains a text file documenting the data framework (Fig. 3a), file naming convention (Fig. 3b), sample metadata, and other details. A table of contents text file describing each experiment alongside sub-directories for each measurement type and supporting processing and visualization scripts. At the time of publication, 113 fabric and 61 fiber specimens are included, as described in the Methods section. The data repository for this work is on the NIST Public Data Repository (PDR) system^25^ and is periodically updated with new versions containing new data from additional instruments, specimens, or characterization methods. The PDR system provides built-in version control, and changes made to the dataset are also tracked via the ChangeLog.txt in the top-level directory.

Under the NIR-SORT top-level directory, the Spectroscopy\ subdirectory contains subfolders for experimental modality (NearInfrared\), instrument (Benchtop_N\, Handheld_F\, or Handheld_S\), data type (Absorbance\ or Reflectance\), and material source (Source_A\, Source_B\, Source_C\, or Source_D\). Sources were defined as origin-defined (Source A and B) or real-world (Source C and D). Source A and B were further differentiated by whether they contain known dyes (Source A) or remain undyed (Source B). Absorbance and reflectance are available for both benchtop and handheld instruments. As mentioned in the Methods section, the daily average temperature and relative humidity of the ambient laboratory conditions were recorded by date in the corresponding TestConditions_N.csv, TestConditions_F.csv, or TestConditions_S.csv files. Descriptions for estimating uncertainties for each instrument are provided under the corresponding instrument subfolder and are described in more detail in the Technical Validation section. For the sample measurements, data are provided as .csv files with formatting native to the “Export to .csv” function in the instrument software, e.g., all data values are provided in scientific notation and significant figures provided are dependent on the instrument resolution. The data do not have column headers: the first column corresponds to x-values, c.f., wavenumbers, and the second column corresponds to y-values, either absorbance or reflectance. For example, in the absorbance folder, all data files will contain wavenumbers in column 1 and absorbance values in column 2. As described in the Methods section, wavenumber can be converted to wavelength via the data_collection_from_folders.py script in the UtilityScripts\ subdirectory.

The Microscopy\ subdirectory contains one instrument, a stereo optical microscope, with three types of sensors (Color, Mono, and Polarized) that were used to image the material sources and produce .bmp or .tiff files. Additional information on the microscope specifications can be found in the README.txt text file in the top-level directory and microscope measurement information can be found in the README_stereo.txt.

All sample files, whether from the spectroscopic or microscopic measurements, follow the same file naming convention (Fig. 3b). Metadata hard-coded into the file name is extracted from either the SampleInformation.csv file in the top-level directory, or from the folder structure. For the example illustrated in Fig. 3b, the source ID, fiber content, and form factor can be cross-referenced in the SampleInformation.csv file for more details on the sample. The instrument and data format relate to where the data file is located within the folder structure and are determined from the experimental parameters. The miscellaneous characters here refer to specimen batch number; however, they can also refer to other variables defined in DataDictionary.csv and are important for the extensibility of the dataset. Metadata not captured in the file name, such as fabric color, dye type, or finish, are available in the SampleInformation.csv.

## Technical Validation

This dataset consists of data from a commercially available benchtop NIR-FTIR spectrometer with performance verification following ASTM E1421^26^ and two commercially available handheld NIR spectrometers built from off-the-shelf components. The benchtop has an internal reference whereas the handhelds do not. To relate the measurements between fabric samples and between instruments, an external reference was used. This external reference was also used to calculate absorbance from handheld reflectance measurements. The following section describes how total expanded uncertainty was estimated for the benchtop and handheld spectrometer measurements.

### Benchtop Estimated Uncertainty

All intensity values are relative to the internal diffuse gold reference. For absolute values, refer to the Fluorilon 99% reflectance target (99W) from Avian Technologies LLC (New London, NH, USA) spectra. See the “README” tab in UncertaintyPropagation_N.xlsx in the Benchtop_N\ directory for a complete explanation and the “99W Raw + Uncertainty” tab for raw, mean, and standard deviation measurement data and expected values for the 99% reflectance standard. For 99W, all coefficients of variation were less than one percent (<1%) of the mean reflectance. In the EstimatedUncertainties_N.xlsx file, data are included from the 99W measured with the internal diffuse gold reference seven times over four dates. The mean and standard deviation were calculated based on the measured values and plotted against the expected values. Four wavelengths were chosen at a 500 nm interval to span the breadth of the NIR range. The calculated Coefficient of Variation for the measured reflectance at these wavelengths, Table 3, show low to negligible variation in the 99% reflectance measurements. This confirms that the benchtop spectrometer can make precise and stable measurements relative to the internal reference.

**Table 3:** Uncertainty estimation for benchtop NIR spectrometer using 99% reflectance standard.

Selected wavelength [nm]	Reflectance mean [%]	Reflectance standard deviation [%]	Coefficient of variation [% of mean]
900	85.0	0.53	0.62
1400	82.8	0.19	0.23
1900	82.1	0.12	0.14
2400	77.2	0.10	0.13

**Fig. 4:**
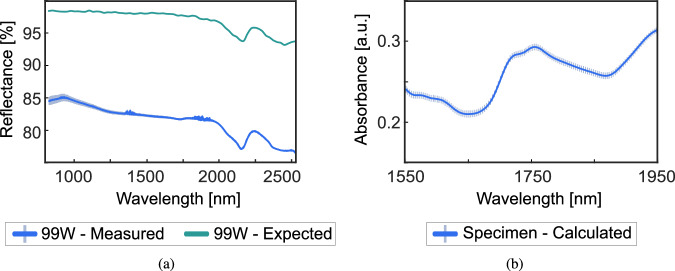
Example uncertainty estimation using the 99% reflectance standard for (**a**) reflectance measurement on benchtop NIR spectrometer, and (**b**) absorbance calculation on handheld NIR spectrometer. The handheld example includes the uncertainty propagation onto sample A39_c100x000x000_00_01_S.csv. Due to the dense sampling of wavelength for the benchtop measurement, error bars overlap; error bars represent one standard deviation.

Figure 4a shows a small standard deviation of 99W measurement with a larger spread at lower wavelengths. The 99W measurement is systematically lower than the known reflectance values of 99W as the measurement of 99W is relative to the internal diffuse gold reference. Therefore, all fabric sample measurements are also relative to the internal diffuse gold reference.

### Handheld Uncertainty Estimation

Absorbance was calculated for Handheld_F and Handheld_S based on the manufacturers’ procedures. Here we describe the procedure for Handheld_S. For Handheld_F procedure and uncertainty estimation, see the corresponding tabs specified below in EstimatedUncertainties_F.xlsx in Handheld_F\. For Handheld_S, see the “README” tab in EstimatedUncertainties_S.xlsx in Handheld_S\ for a complete explanation and “Estimation of Uncertainty” tab for an uncertainty calculation for an example spectrum. From the “README” tab of the EstimatedUncertainties_S.xlsx file, each sample was collected with a dark, i.e., light source off, and light, i.e., light source on, measurement denoted as such in Column A. A 99% reflectance standard specimen was measured three times at the beginning (Columns C, D, E) and three times at the end (Columns QV, QW, QX) of the sample collection. The raw reflectance data is used to calculate absorbance as a function of wavelength with Equation (1),

1 $$Absorbance(\lambda )=-{\log }_{10}\frac{{S}_{light}(\lambda )-{S}_{dark}(\lambda )}{{S}_{ref}(\lambda )-{S}_{dark}(\lambda )}$$

where *λ* is the wavelength, *S*_*light*_ is the spectrum from the light measurement, *S*_*dark*_ is the spectrum from the dark measurement, and *S*_*ref*_ the spectrum from the 99% reflectance standard.

The propagated uncertainty as a function of wavelength (Column N), derived in the Supplementary Information, is estimated from repeat measurements of the 99% reflectance standard specimen and sample specimen using Equation (2):

2 $$\Delta Absorbance(\lambda )=\sqrt{{(\frac{\Delta {S}_{light}}{C\ast ({\overline{S}}_{light}-{\overline{S}}_{dark})})}^{2}+{(\frac{\frac{{\overline{S}}_{light}-{\overline{S}}_{ref}}{{\overline{S}}_{ref}-{\overline{S}}_{dark}}\ast \Delta {S}_{dark}}{C\ast ({\overline{S}}_{light}-{\overline{S}}_{dark})})}^{2}+{(\frac{\Delta {S}_{ref}}{C\ast ({\overline{S}}_{ref}-{\overline{S}}_{dark})})}^{2}}$$

where C = $\mathrm{ln}(10)\approx$ 2.30259 and overbars represent the mean of the variable taken over six repeat measurements. Values for the reference mean and standard deviation as a function of wavelength can be found in Columns B and E, respectively. Reference values do not change between samples. Values for the light and dark mean and standard deviation are sample dependent and can be found in Columns C, D, F, and G, respectively. An example plot of the mean calculated absorbance (column M) of a specific sample with error bars representing the estimated uncertainty is shown in Fig. 4b.

## Usage Notes

Among other applications, NIR-SORT can be used in the development of NIR systems to inform hardware decisions and test or validate ML models. For example, benchtop data can be used as a high quality benchmark in peak importance analyses to determine optimal spectrometer wavelength ranges depending on fiber(s) of interest. They can also be used during feature engineering to confirm whether differences detected in discriminant analyses are real or signal artifacts of the user’s data collection. Both handheld and benchtop data can be used to test and validate ML models, to test and understand the effects of preprocessing, postprocessing or other data treatments, and for other algorithm development tasks. Beyond system and model development, NIR-SORT could also provide a baseline to investigate potential effects or artifacts from material treatments or conditions, such as fabric color, wear, and washing. While the initial version of NIR-SORT uses fiber content information from Source C’s garment care labels and Source D’s product sample cards, we are in the process of cross-validating these fiber compositions and will provide an update in the next version. In the meantime, we do not recommend using Source C and D for training purposes and rather recommend using the real-world samples’ spectra for exploration purposes.

NIR-SORT is designed to be human-readable, human-actionable as well as annotated and ready to be ingested for ML and artificial intelligence workflows. For data to be digested into a machine-readable, machine-actionable format, the data_collection_from_folders[.sh, .py] script, available as a Bash or Python script, collects data files from specified subfolders and copies them to a single combined DataGrabs\ folder or retains the data in memory. Subsequent scripts can either read in files from this single folder or call the collection as a function and use grabbed data in memory. Four other example tasks are included: converting wavenumber to wavelength and writing out to a new file (data_collection_from_folders[.py, .sh]), plotting spectra based on filename metadata (example_basic_data_visualization.py), plotting interpolated Benchtop_N spectral data with Handheld_S data for comparison (example_benchtop_to_handheld_comparison.py), and plotting a user defined subset of spectra based on metadata in SampleInformation.csv (example_color_comparison.py).

### Data visualization

**Fig. 5:**
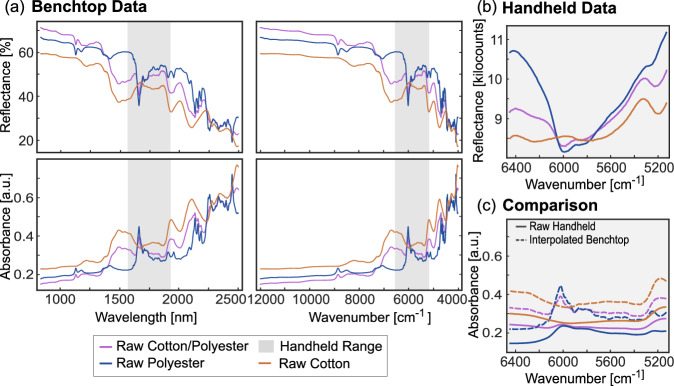
Example data from one replicate of each of a cotton, polyester, and nominally 50% cotton/50% polyester blend collected with the Benchtop_N and Handheld_S spectrometers. (**a**) Shows the example benchtop data plotted for each combination of reflectance, absorbance, wavelength and wavenumber with grey shaded area representing the respective handheld range. (**b**) Shows the reflectance vs wavenumber data from the handheld for the same specimens. Note that the handheld reflectance is reported in detector counts rather than the % reflectance unit of the benchtop. (**c**) A comparison of handheld absorbance data to benchtop absorbance resampled at the wavenumbers used by the handheld with a cubic spline interpolant. Error bars are omitted for clarity of presentation, for uncertainty information please refer to the uncertainty subsection.

NIR-SORT is designed for multiple types of users, and the example scripts help orient users depending on their needs. The textile research community typically works with absorbance spectra, whereas it is more common for reflectance to be collected and analyzed in the field. The basic data visualization example script (example output of which is shown in Fig. 5a) is an example of accessing and plotting the absorbance vs reflectance format and performing a wavenumber vs wavelength conversion. Similarly, handheld data can be read in and plotted, but units may differ (e.g., see Fig. 5b). As an example of comparing the handheld instrument results to the benchtop, handheld data from Handheld_S is plotted as received with benchtop data interpolated with a bicubic spline onto handheld wavelengths on the same axes in Fig. 5c. The Python script example_benchtop_to_handheld_comparison.py is an example of this workflow. Figure 5 was plotted using code from these Python scripts in Visual Studio Code with script execution plug-ins and a Windows Subsystem for Linux (WSL) terminal running Ubuntu 22.04 to function as an integrated development environment (IDE) with Seaborn and Matplotlib data visualization packages. Since data is provided in a simple .csv format, dataset users can straightforwardly use other IDEs, packages, or programming language as needed. In addition to the basic data visualizations shown in Fig. 5 that compare material source and fiber content, comparing other characteristics such as fabric color can be important. The Python script example_metadata_comparison.py shows an example of cross-referencing the metadata file (SampleInformation.csv) to extract data files for fabrics’ specific characteristics – in this example script, color – to compare (viz. black versus red).

### Data processing

**Fig. 6:**
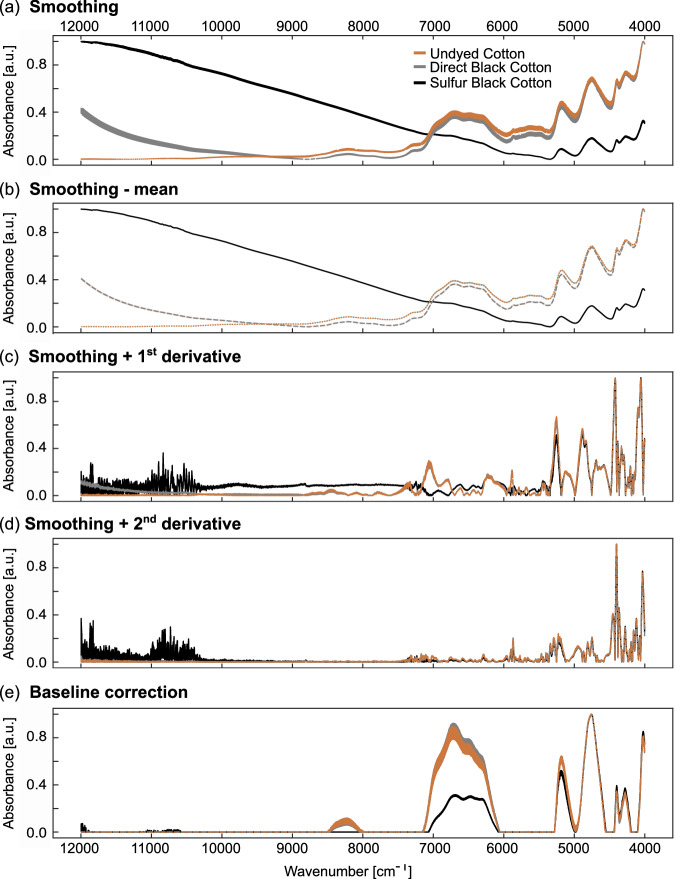
Example data from seven replicates of each of an undyed cotton, direct black dyed cotton, and sulfur black dyed cotton collected with the benchtop spectrometer and processed with Open Specy analysis program. (**a**) min-max normalize and Savitzky-Golay (SG) filter, no estimator, (**b**) min-max normalize and SG filter, mean estimator, (**c**) min-max normalize, SG filter, first derivative, (**d**) min-max normalize, SG filter, second derivative, and (**e**) min-max normalize, baseline correction. Error bars are omitted for clarity of presentation, for uncertainty information please refer to the uncertainty subsection.

Reduced data can be further processed to prepare for use in regression or classification models with various resources such as proprietary software packages included with a benchtop spectrometers or open source tools such as Open Specy^27^. Here we provide an example of typical processing methods using Open Specy by importing the files to the web application, selecting the corresponding processes, and exporting the processed data. The example results are plotted in Fig. 6. Typical processing pipelines include normalization, smoothing, derivatives and baseline correction^28^. Depending on what baseline correction is applied, information that is pertinent to building robust ML models can be lost. In this case, we used the IModPolyFit function for a polynomial baseline correction. In the example in Fig. 6e, the signals at the near-visible end of the spectrum were incorrectly assigned as baseline drift and removed during the baseline correction. These signals are not noise, signal artifacts, or baseline drift: in this example they correspond to dyes. Losing this information could potentially lead to misrecognition of the material composition. Baseline offsetting errors can be minimized instead by analyzing the first-derivative (Fig. 6c) or second-derivative (Fig. 6d) of the spectra.

### Data Interfaces

#### NIST PDR interface

NIST provides a data repository service, the PDR, that is automatically linked to data.gov, the official source for all U.S. Government open science data. The PDR provides an interface to access and download NIR-SORT data^25^. The landing page presents the user with an abstract and overview of the dataset, a file browser, and metadata information downloadable in a JSON-like format known as NERDm. An interface element allows the user to link to a bulk-data download information page that includes a pre-generated Python script for automated downloading of data. For manual downloading, the file browser uses a tree table to view the directory structure and files. This structure is as described above and shown in Fig. 3.The tree table contains columns for the file or folder name under “Name” column, the type of data under “File Type” column, the size under “Size” column, and download status under “Access” column. Users can download files either via direct download for individual files by clicking on the down-arrow symbol or via the add-to-cart feature for multiple files or complete directories by clicking on the cart symbol. Once desired files or directories are selected, the user clicks on the cart icon at the top right corner of the webpage, which brings them to Download Manager page where their selections can be change. The user can click “Download Selected” to download to a .zip archive.

#### Open Specy

NIR-SORT is also available via the Open Specy web application as an internal reference library. The data can be downloaded as test data and are used in the “Identification” algorithm that returns material composition predictions for sample data via correlation-based matching criteria. For more information on using the Open Specy interface see Cowger *et al*.^27^ and the related documentation.

#### Materials Data Facility (MDF)

NIR-SORT is also available via a MDF repository^29^. The full dataset can be downloaded as a .zip file or individual files can be downloaded via the Globus File Manager web application. To download multiple files or directories, users can install a free Globus Personal endpoint to establish a transfer between their storage location and MDF. For more information on using the MDF interface see Blaiszik *et al*. 2016^30^ and the related documentation.

## Supplementary information

Supplementary Information

## Data Availability

The Near Infrared Spectra of Origin-defined and Real-world Textiles (NIR-SORT) dataset is available to view and download from https://data.nist.gov/od/id/mds2-3325 (PDR) or 10.18126/61v0-rf98 (MDF). Supplemental data and scripts are available to view and download from 10.18434/mds2-4128 (PDR). The complete data and code packages are released free and open source per the terms of the NIST license included in the root directory.

## Code availability

The data handling and post processing scripts, with the exception of those built into Open Specy, are available in the data repository folder UtilityScripts\. The Open Specy analyses were conducted using the tools available via the web application as described above. The Python scripts were written and tested on Windows 10 x64 using Visual Studio Code v1.9 with a WSL Ubuntu 22.04 terminal or Spyder 5 with Python version 3.9.18, and packages Numpy (1.26.4), Scipy (1.11.4), Pandas (1.3.4), Seaborn (0.12.2), and Matplotlib (3.5.0). The bash script was run with GitBash 5.2.26 via a Visual Studio Code terminal. See the README_for_scripts.txt and HowToRun.txt text files contained in the UtilityScripts\ subdirectory for details.

The complete data and code packages are released free and open source per the terms of the NIST license included in the root directory.
