# Supplementary material for: A spectroscopic dataset for known provenance and post-consumer textiles
Source: Sci Data. 2026 May 22;13:1188. doi: 10.1038/s41597-026-07434-6 (PMC13473549; doi:10.1038/s41597-026-07434-6)
Supplement: Supplementary file 1 [file 41597_2026_7434_MOESM1_ESM.pdf]

Supplemental Information for: A spectroscopic dataset for known provenance and post-consumer textiles

Katarina E. Goodge (0000-0001-9262-4125)[1,2,*], Alexander K. Landauer (0000-0003-2863-039X)[1], Cecelia Vederman (0009-0009-5343-1545)[1,3], and Amanda L. Forster (0000-0001-7397-4429)[1,*]

[1]National Institute of Standards and Technology, Material Measurement Laboratory, 100 Bureau Drive, Gaithersburg, MD 20899
[2]Georgetown University, Institute for Soft Matter Synthesis and Metrology, 3700 O St NW, Washington, DC 20057
[3]Montgomery College, 20200 Observation Drive, Germantown, MD 20876
[*]Corresponding authors: Katarina Goodge (katarina.goodge@nist.gov) and Amanda Forster (amanda.forster@nist.gov)

ABSTRACT

Near infrared (NIR) spectroscopy is a rapid, non-invasive technique often used for chemical bond structure identification, making it a prime candidate for feedstock identification and validation for industrial processes involving polymers. It has rapidly gained popularity in the textile industry; however, the availability of high-quality, known provenance NIR data for textile fibers and fabrics is limited. Applying NIR to answer questions such as fiber classification or polymer blend identification typically requires the use of models or algorithms. The underpinning data for these models is typically in proprietary libraries or self-built databases; thus, benchmarking model performance across the industry is challenging. Here, a new dataset is presented to address this challenge. The dataset contains data on textile specimens for applications including fiber content classification, systems and software development, and validation of textile sorting systems. The data repository includes directories for benchtop NIR spectral data, handheld NIR spectral data, and fabric-scale microscopy images.

Supplemental Data

To recreate Figure 5, Figure 6, and Figure S1, scripts and associated data are available to download from NIST Public Data Repository: https://doi.org/10.18434/mds2-4128. Please see the README file within the PDR for more information.

Supplemental Figures

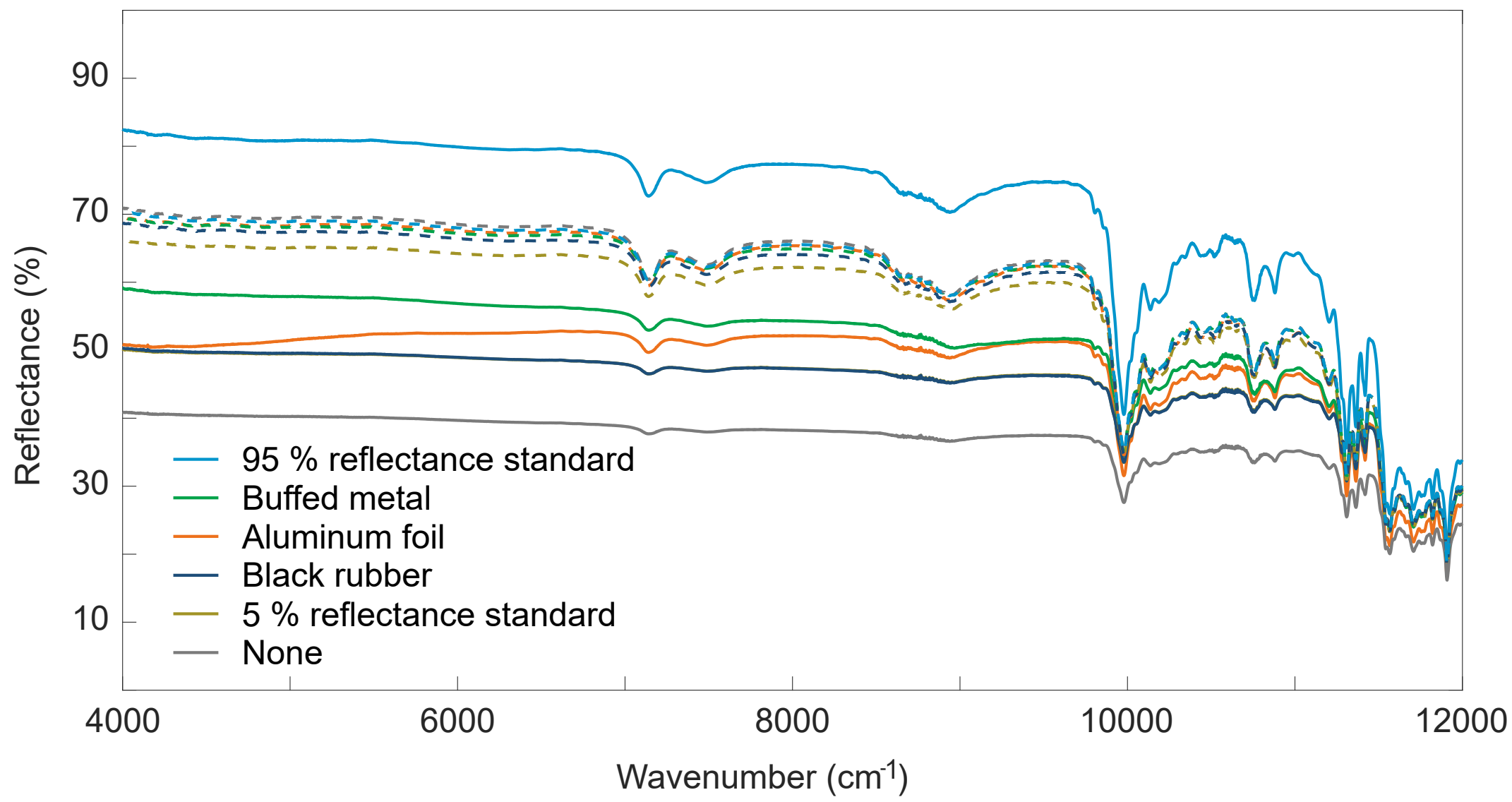

Figure S1: NIR spectra of one layer (solid) and six layers (dashed) of a woven polyester (B09) specimen with example backing materials.

Uncertainty Propagation Derivation

For Handheld_S, absorbance is calculated from reflectance measurements with light, dark, and reference:

$$Absorbance(\lambda) = -\log_{10} \frac{S_{light}(\lambda) - S_{dark}(\lambda)}{S_{ref}(\lambda) - S_{dark}(\lambda)}$$

where λ is the wavelength, S_{light} is the spectrum from the light measurement, S_{dark} is the spectrum from the dark measurement, and S_{ref} the spectrum from the 99 % reflectance standard.

$$\Delta Absorbance(\lambda) = \sqrt{\left(\frac{\partial Absorbance}{\partial S_{light}} \Delta S_{light}\right)^2 + \left(\frac{\partial Absorbance}{\partial S_{dark}} \Delta S_{dark}\right)^2 + \left(\frac{\partial Absorbance}{\partial S_{ref}} \Delta S_{ref}\right)^2}$$

$$\Delta Absorbance(\lambda) =$$
$$\sqrt{\left(\frac{1}{(S_{light} - S_{dark}) * ln(10)} \Delta S_{light}\right)^2 + \left(\frac{S_{light} - S_{ref}}{(S_{ref} - S_{dark})(S_{light} - S_{dark}) * ln(10)} \Delta S_{dark}\right)^2 + \left(\frac{1}{(S_{ref} - S_{dark}) * ln(10)} \Delta S_{ref}\right)^2}$$

$$\Delta Absorbance(\lambda) = \sqrt{\left(\frac{\Delta S_{light}}{C * (\bar{S}_{light} - \bar{S}_{dark})}\right)^2 + \left(\frac{\frac{\bar{S}_{light} - \bar{S}_{ref}}{\bar{S}_{ref} - \bar{S}_{dark}} * \Delta S_{dark}}{C * (\bar{S}_{light} - \bar{S}_{dark})}\right)^2 + \left(\frac{\Delta S_{ref}}{C * (\bar{S}_{ref} - \bar{S}_{dark})}\right)^2}$$

where C = ln(10) $\approx$ 2.30259
